# Cooperate or Not Cooperate in Predictable but Periodically Varying Situations? Cooperation in Fast Oscillating Environment

**DOI:** 10.1002/advs.202001995

**Published:** 2020-09-18

**Authors:** S. G. Babajanyan, Wayne Lin, Kang Hao Cheong

**Affiliations:** ^1^ Science, Mathematics and Technology Cluster Singapore University of Technology and Design 8 Somapah Road S487372 Singapore; ^2^ SUTD‐Massachusetts Institute of Technology International Design Centre S487372 Singapore

**Keywords:** dynamical systems, game theory, nonlinear dynamics, population dynamics, prisoner's dilemma

## Abstract

In this work, the cooperation problem between two populations in a periodically varying environment is discussed. In particular, the two‐population prisoner's dilemma game with periodically oscillating payoffs is discussed, such that the time‐average of these oscillations over the period of environmental variations vanishes. The possible overlaps of these oscillations generate completely new dynamical effects that drastically change the phase space structure of the two‐population evolutionary dynamics. Due to these effects, the emergence of some level of cooperators in both populations is possible under certain conditions on the environmental variations. In the domain of stable coexistence the dynamics of cooperators in each population form stable cycles. Thus, the cooperators in each population promote the existence of cooperators in the other population. However, the survival of cooperators in both populations is not guaranteed by a large initial fraction of them.

## Graphical abstract

The cooperation problem between two populations in a periodically varying environment—in the context of the two‐population prisoner's dilemma game with periodically oscillating payoffs—is discussed. New nonlinear dynamics are discovered, and nonzero levels of cooperation emerge as possible outcomes under certain conditions on the environmental variations. The dynamics in these regions of stable coexistence of cooperators form stable cycles.

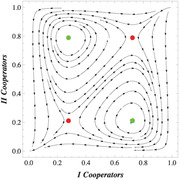

## 1 Introduction

Understanding the mechanisms behind the emergence of cooperation between different agents is of great interest. This problem has produced a vast literature, and remains one of the major focuses of different scientific paradigms.^[^ ^1^, ^2^, ^3^, ^4^, ^5^, ^6^, ^7^, ^8^, ^9^, ^10^, ^11^, ^12^, ^13^, ^14^, ^15^ ^]^ Cooperation of agents generates a common good for all interacting agents, including those who do not cooperate. However, cooperative behavior is associated with a cost of cooperation, and so cooperative behavior is assumed to be unfavorable for interacting agents in the absence of supporting mechanisms. Famous examples of obstacles in cooperation problems are the prisoner's dilemma game^[^ ^16^, ^17^, ^18^ ^]^ and the tragedy of the commons game.^[^ ^19^ ^]^ The prisoner's dilemma game describes a situation faced by two agents who can choose between two possible strategies, cooperation or defection. If both players cooperate, then they will receive a higher payoff compared to the mutual defection situation. However, cooperation between rational agents is impossible due to the structure of the game. The impossibility of the emergence of cooperation between players is described by well‐known solution concepts of game theory, that is, Nash equilibrium or dominated strategy analysis. Evolutionary game theory (EGT) extends the usual game‐theoretic concepts, which are mostly static, to model the dynamical behavior (i.e., the ongoing selection process) in different populations.^[^ ^3^, ^10^, ^20^, ^21^ ^]^ Various models have been suggested for describing the underlying mechanisms of the emergence of cooperation in selection processes.^[^ ^5^, ^22^, ^23^, ^24^, ^25^, ^26^, ^27^, ^28^, ^29^, ^30^ ^]^ These mechanisms are based on different phenomena such as structured population, relatedness, direct and indirect reciprocity.

Our analysis is based on replicator dynamics (RD),^[^ ^3^, ^20^, ^21^, ^31^ ^]^ an important model for the selection process. RD describes the change of different types within a population due to the change of the frequency‐dependent fitnesses of these types. The fitnesses of various types are defined by the pairwise interaction coefficients (payoff matrix). Therefore, the prediction of RD for the prisoner's dilemma game in the fixed environment—where the pairwise interaction coefficients do not vary—is the same as for the traditional game theoretic analysis: the selection process does not favor cooperation.

It is known that the evolutionary processes in varying environments can drastically differ from those in a fixed environment.^[^ ^32^, ^33^, ^34^, ^35^, ^36^, ^37^, ^38^, ^39^, ^40^, ^41^, ^42^, ^43^, ^44^, ^45^, ^46^, ^47^, ^48^, ^49^, ^50^, ^51^ ^]^ A changing environment can induce polymorphic states that do not exist in a fixed environment.^[^ ^34^, ^37^, ^39^, ^41^, ^52^, ^53^, ^54^, ^55^, ^56^, ^57^, ^58^ ^]^ Here, we are going to discuss the selection process in well‐mixed populations under a periodically varying environment. Periodic variations in the environment can be associated with annual seasonal variations for animals and/or the nutrient supply for cells.^[^ ^32^, ^59^, ^60^, ^61^ ^]^

In this paper, periodic variations in the environment are incorporated into our model as a periodic change in the pairwise interaction coefficients. We further assume that the environment goes through multiple oscillations during the intra‐population selection process. Hence, timescale separation is assumed: the fast timescale is associated with the period of environmental variations, while the slow timescale represents the evolutionary dynamics of the population. The pairwise interaction coefficients are composed of constant and periodically oscillating parts: the constant parts satisfy the conditions imposed by the prisoner's dilemma game, and the time‐average of the oscillating parts over a period of environmental variations vanishes. However, the possible overlaps of time‐varying coefficients cause new nonlinear terms in the evolutionary dynamics of the populations. Such evolutionary dynamics can lead to the survival of a cooperators in each population.

The paper is organized as follows. We first discuss the two‐population replicator dynamics in different biological settings. Next, we discuss some illustrative examples of the possible dynamical effects induced by the periodically varying environment in a two‐population prisoner's dilemma game. Finally, we give some possible extensions to this work.

## 2 Model

### 2.1 Two‐Population Evolutionary Dynamics and Prisoner's Dilemma Game

Let us briefly recall the two‐player prisoner's dilemma game, in which the problem of cooperation is illustrated. Each player has two strategies: cooperate (c) or defect (d). The payoffs of the players for every possible chosen pair of strategies are depicted in the following matrix.

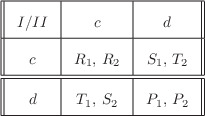



Here, it is assumed that $T_{i}>R_{i}>P_{i}>S_{i},\;i=1,2$. From the ordering of the payoffs, it follows that each agent prefers defection rather than cooperation since the cooperation strategy (c) is dominated by the defection strategy (d): no matter what the opponent does, defection provides higher payoff than cooperation. Indeed, this situation—where both agents defect—is the only Nash equilibrium of the game, that is, a situation in which no player will gain by unilaterally changing its strategy. So the game ends up in the situation where both players defect. However, from the ordering of the payoffs, it follows that if both agents would choose the cooperation strategy instead, then the outcome of the game would be preferable to both agents.

Let us now denote the frequencies of the cooperators in each population respectively by p and q. (Frequencies of the defector type in populations I and II will be 1−p and 1−q, respectively: this follows from normalization.) It is known that the dynamical properties of the system described by replicator equations (see Supporting Information) remain invariant under subtraction of constant quantities from the columns and rows.^[^ ^20^ ^]^ This property allows us to present the payoff matrix in the form

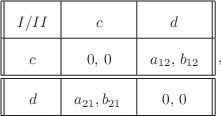
where $a_{12}=S_{1}-P_{1}$, $a_{21}=T_{1}-R_{1}$, $b_{12}=T_{2}-R_{2}$ and $b_{21}=S_{2}-P_{2}$. Moreover, from the ordering of the payoffs it follows that $a_{12}<0<a_{21}$ and $b_{21}<0<b_{12}$. In this case mutual cooperation and mutual defection both provide the same fitness (payoff); however, as mentioned above the dynamical properties of the replicator dynamics remains the same. After this simplification, the replicator dynamics of the two‐population, two‐type situation attain the following form:

(1) $$\frac{dp}{dt}=p\left(1-p\right)\left(a_{12}-\left(a_{12}+a_{21}\right)q\right)$$

(2) $$\frac{dq}{dt}=q\left(1-q\right)\left(b_{21}-\left(b_{12}+b_{21}\right)p\right)$$

It should be noted that here the normalization of the frequencies are taken in account,^[^ ^3^, ^20^ ^]^ so the given dynamical system is defined on the simplex $S^{1}\times S^{1}$. Taking into account the orderings between the simplified payoffs it is seen that the dynamical system (1)‐(2) does not admit a rest point in the interior of the $S^{1}\times S^{1}$ simplex. Moreover, it can be shown that the only stable rest point is the vertex of the simplex which corresponds to mutual defection (p,q)=(0,0) (see Supporting Information). Thus, all trajectories starting from a point in the interior of the simplex converge to the stable mutual defection vertex.

### 2.2 Evolutionary Dynamics in Fast Periodically Varying Environment. New Dynamical Terms in Two‐Population Replicator Dynamics Due to Environmental Variations

Here we focus on the description of the behavior of the system (1)‐(2) in the presence of a varying environment. Environmental variations are incorporated as fast and periodic changes in the pairwise interaction coefficients (i.e., the payoff matrix). These coefficients become periodic functions with period 2π/ω, i.e. $a_{ij}\left(\tau \right)=a_{ij}\left(\tau +2\pi \right)$ and $b_{kl}\left(\tau \right)=b_{kl}\left(\tau +2\pi \right)$, where τ=ωt is a fast timescale (i.e., the timescale in which environmental variations occur), and ω>1 is the frequency of the periodic environmental variations.

We separate the time‐dependent payoffs into constant and oscillating parts as follows:

(3) $$a_{ij}\left(\tau \right)={\bar{a}}_{ij}+{\tilde{a}}_{ij}\left(\tau \right),\;\;b_{ij}\left(\tau \right)={\bar{b}}_{ij}+{\tilde{b}}_{ij}\left(\tau \right)$$

The time‐averages of the oscillating parts over a period of environmental variations are zero.

(4) $$\bar{{\tilde{a}}_{ij}}\left(\tau \right)\equiv \int_{0}^{2\pi }\frac{d\tau }{2\pi }{\tilde{a}}_{ij}\left(\tau \right)=0$$

(Note that the same condition holds for ${\tilde{b}}_{kl}$.) Let us introduce also the primitives of the oscillating payoff coefficients, ${\hat{a}}_{ij}$ and ${\hat{b}}_{ij}$, via the equations $\partial_{\tau }{\hat{a}}_{ij}={\tilde{a}}_{ij}$ and $\partial_{\tau }{\hat{b}}_{ij}={\tilde{b}}_{ij}$. It is obvious that these quantities vary by the same period and average to zero over a period of oscillation.

We follow the Kapitza method proposed initially for the problem of the rigid pendulum with an oscillating point of suspension.^[^ ^62^, ^63^ ^]^ We represent the frequency of cooperators in population I as a slowly varying part $\bar{p}$ (i.e., a part that is averaged over a period of environmental variation) and an oscillating part ε, and likewise split the frequency of cooperators in population II into a slow‐varying part $\bar{q}$ and an oscillating part η. The oscillating parts vary on the fast timescale and average to zero over a period of environmental changes. We assume that each of the oscillating terms, that is, ε and η, depend on the slow‐varying frequencies of both populations, $\bar{p}\left(t\right)$ and $\bar{q}\left(t\right)$.

(5) $$p\left(t\right)=\bar{p}\left(t\right)+\varepsilon \left(\bar{p},\bar{q},\tau \right),\;q\left(t\right)=\bar{q}\left(t\right)+\eta \left(\bar{p},\bar{q},\tau \right)$$

(6) $$\int_{0}^{2\pi }\varepsilon \left(\bar{p}\left(t\right),\bar{q}\left(t\right),\tau \right)\frac{d\tau }{2\pi }=\int_{0}^{2\pi }\eta \left(\bar{p}\left(t\right),\bar{q}\left(t\right),\tau \right)\frac{d\tau }{2\pi }=0$$

Putting (5) into Equations (1) and (2), we obtain a system of equations which consist of the quantities varying on the different timescales (see Supporting Information for the derivation details). From these, we will be able to determine the behavior of the frequencies of different types in various timescales.

Let us provide here some sketch of the derivation steps. We first expand the right hand‐sides of the system (1)‐(2) over the oscillating parts $\varepsilon (\bar{p}\left(t\right),\bar{q}\left(t\right),\tau )$ and $\eta (\bar{p}\left(t\right),\bar{q}\left(t\right),\tau )$. The oscillating parts are searched for via expanding over $\frac{1}{\omega }$, where ω is the frequency of the periodic variations of the environment. Then we separate the quantities which vary on fast timescale τ and are of order O(1). In this step we determine the dynamics of the oscillating parts. The behavior of these parts are defined by the system similar to the system (1)‐(2), where in the right hand sides only slow‐varying frequencies are present and the payoffs are ${\hat{a}}_{ij}$ and ${\hat{b}}_{ij}$.

After this, we average the remaining part of the system over a period of environmental changes keeping quantities up to $O(1/\omega^{2})$. In this way, we obtain the slow‐time variations of the frequencies of different types in each population.

For the considered problem we do a further simplification by assuming that ${\bar{a}}_{12}=-{\bar{a}}_{21}=a<0$ and ${\bar{b}}_{21}=-{\bar{b}}_{12}=b<0$. Note that these assumptions are imposed only on the constant parts. In terms of the payoffs of the prisoner's dilemma game those assumptions will be ${\bar{T}}_{1}+{\bar{S}}_{1}={\bar{R}}_{1}+{\bar{P}}_{1}$ and ${\bar{T}}_{2}+{\bar{S}}_{2}={\bar{R}}_{2}+{\bar{P}}_{2}$.

The slow‐time (averaged over the environmental variations) varying frequencies can then be determined from the following system of equations:

(7) $$\frac{d\bar{p}}{dt}=\bar{p}\left(1-\bar{p}\right)\left(a-\bar{q}\left(1-\bar{q}\right)\left(\beta -\alpha \bar{p}\right)\right)$$

(8) $$\frac{d\bar{q}}{dt}=\bar{q}\left(1-\bar{q}\right)\left(b-\bar{p}\left(1-\bar{p}\right)\left(\gamma +\alpha \bar{q}\right)\right)$$

where the Greek letters denote the following quantities:

(9) $$\begin{array}{cc} \beta \equiv & \frac{1}{\omega }\bar{{\hat{b}}_{21}\left({\tilde{a}}_{12}+{\tilde{a}}_{21}\right)},\;\;\gamma \equiv \frac{1}{\omega }\bar{{\hat{a}}_{21}\left({\tilde{b}}_{12}+{\tilde{b}}_{21}\right)}\\ & \alpha \equiv \frac{1}{\omega }\bar{\left({\tilde{a}}_{12}+{\tilde{a}}_{21}\right)\left({\hat{b}}_{12}+{\hat{b}}_{21}\right)} \end{array}$$

From (7) and (8) it follows that the slow‐varying frequencies $\left(\bar{p},\bar{q}\right)$ are again defined on the simplex $S^{1}\times S^{1}$. Moreover, it is seen that the stability of the vertices of $S^{1}\times S^{1}$ remain unchanged by the environmental variations: this is due to the fact that the newly generated non‐linear terms and their gradients vanish at each vertex (see Supporting Information for details). In particular, the mutual defection point still remains as a stable state for the slow‐varying frequencies.

It is seen from the slow‐varying dynamics that the behavior of a given population depends on both its own payoffs and the payoffs of the opponent, while in the fixed environment two‐population replicator dynamics (1)‐(2) the behavior of a given population depends only on its own payoffs. These new nonlinear terms are based on the overlaps of different payoffs in one period of environmental variation.

From the definition of the nonlinear payoffs (9) it is obvious that these payoffs will vanish if all pairwise interaction coefficients vary by the same periodic function ${\tilde{a}}_{ij}\propto \xi \left(\tau \right)$ and ${\tilde{b}}_{ij}\propto \xi \left(\tau \right)$, where ξ(τ+2π)=ξ(τ). The contribution from the environmental variation will be non‐trivial if the oscillating parts of at least two coefficients vary in different phase.

## 3 Results

Here we discuss some illustrative examples of the possible dynamical effects induced by the periodically varying environment in a two‐population prisoner's dilemma game. Note that while environmental variations change the pairwise interaction coefficients, the time‐average of these coefficients over a period of environmental variations form the prisoner's dilemma game. Thus, at any given moment the payoffs' ordering can differ from the ordering of prisoner's dilemma game. It is obvious also, that if at every given moment the payoffs satisfy the ordering imposed by the prisoner's dilemma game, then there will be no new dynamical effect.

Let us recall the imposed conditions over the pairwise interaction coefficients. We assume that ${\bar{T}}_{1}+{\bar{S}}_{1}={\bar{R}}_{1}+{\bar{P}}_{1}$ and ${\bar{T}}_{2}+{\bar{S}}_{2}={\bar{R}}_{2}+{\bar{P}}_{2}$. In this case, the diagonal terms of the payoff matrix are nullified. We introduced further notation as follows: ${\bar{a}}_{12}=-{\bar{a}}_{21}=a<0$ and ${\bar{b}}_{21}=-{\bar{b}}_{12}=b<0$. (The inequality is a result of the ordering condition.)

Introducing periodic variation into the pairwise interaction coefficients can create new dynamical effects. Of these we know that the stability of the vertices of $S^{1}\times S^{1}$ remain unchanged, and no new rest points form on the boundary of the simplex (see Supporting Information). We also know that new rest points may emerge in the interior of the simplex, and that they will emerge as center‐saddle pairs (see Experimental Section). For the α=0 case, explicit expressions exist for the new rest points and the conditions under which they form, and it can be shown that the number of non‐degenerate interior rest points will be 0 or 4.

In the rest of this section, we will concurrently discuss the two cases α=0 and α≠0. As we have seen above, for the first case the conditions for appearance of the new rest point are explicit. For the second case, we mostly base our discussion on numerical results. Nevertheless, the nature of new phenomena are the same for both cases.

**Figure 1:**
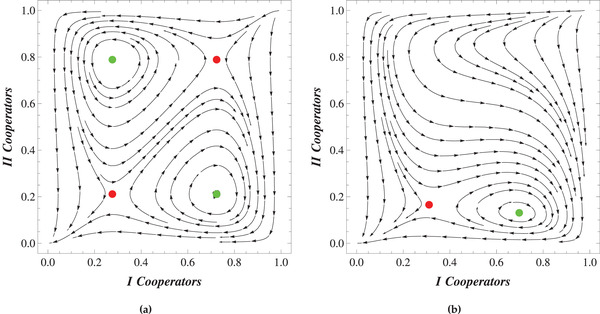
Phase portrait of slow‐varying frequencies. The ordinates show the frequencies of cooperators $\bar{p}$ and $\bar{q}$ in populations I and II. a) Represents the phase space diagram for a=−0.1, b=−0.2, β=−0.6, γ=−1 and α=0. b) Illustrates the phase space portrait for α=0.4, with all other quantities the same. The stable and unstable rest points (centers and saddles, respectively) are depicted respectively by green and red points. The existence of α≠0 narrows the possible initial state space from which the coexistence of different types is possible.

In **Figure** 1 phase space diagrams are illustrated for the dynamical system (7)‐(8) under different sets of parameters. These diagrams illustrate the possible behavior of slow‐varying frequencies started from different initial states. The axes represent the frequencies $\bar{p}$ and $\bar{q}$ of the cooperators in populations I and II averaged over environmental oscillations.

In Figure 1a the phase space diagram is illustrated for the α=0 case. As we note in the Experimental Section, it is possible to create four new non‐degenerate rest points in the interior of $S^{1}\times S^{1}$, and the rest points will be symmetric about the point $\left(\frac{1}{2},\frac{1}{2}\right)$. For this particular illustrative example, the other nonlinear payoffs β and γ have been chosen so that we get four new rest points in the interior of the simplex. The stable (center) and unstable (saddle) rest points are depicted respectively by green and red points. We have shown that in this case the stable rest point which satisfies ${\bar{p}}^{*}>\frac{1}{2},{\bar{q}}^{*}<\frac{1}{2}$ (respectively ${\bar{p}}^{*}<\frac{1}{2},{\bar{q}}^{*}>\frac{1}{2}$) is a local minimum (maximum) of the Hamiltonian function $H(\bar{p},\bar{q})$ given by (11).

In the neighborhood of these rest points the trajectories are closed orbits, which means that stable coexistence of cooperators and defectors in each population is possible in contrast to the fixed environment case.

In Figure 1b a particular example for the α≠0 case is illustrated. In this particular case environmental variations induce only two new rest points in the dynamics: one stable (center) and one unstable (saddle). In contrast to the previous case, here the set of all initial states for which the coexistence of different types is possible is narrower. The only stable rest point corresponds to the local minimum of $H(\bar{p},\bar{q})$. It should be noted that it is also possible in the α≠0 case to produce four instead of two new rest points: for example, if we were to take the parameters used for Figure 1a, then increase α by a sufficiently small amount.

Note that all the trajectories which are not closed orbits, with the exception of the stable manifolds of the interior saddle points, go to the mutual defection point. This follows from the fact that environmental variations do not alter the stability of the vertices of $S^{1}\times S^{1}$. Hence, the mutual defection point remains as a stable state of the replicator dynamics.

**Figure 2:**
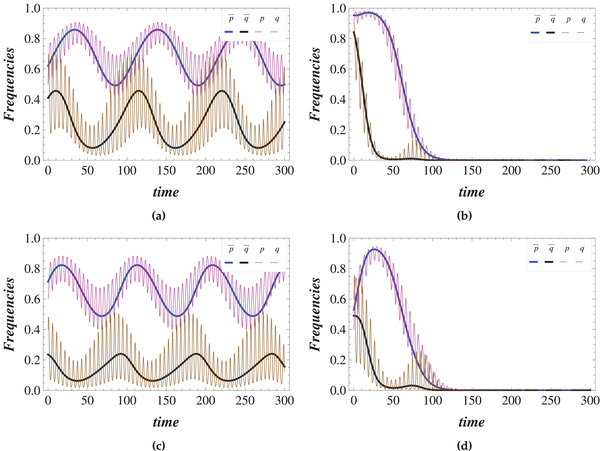
Dynamics of slow‐varying (averaged) $\left(\bar{p},\bar{q}\right)$ and non‐averaged (p,q) frequencies of cooperators in populations I and II. The magenta (brown) line illustrates the non‐averaged frequency of cooperators in population I (II). The smooth blue (black) line is the averaged proportion of cooperators $\bar{p}$ ($\bar{q}$). The parameters of the models are chosen as follows: a=−0.1, b=−0.2, β=−0.6, γ=−1, ω=1.2. a,b) Correspond to α=0 case. The initial conditions for frequencies of cooperators in (a,b) are respectively $p_{0}=0.5$, $q_{0}=0.5$ and $p_{0}=0.9$, $q_{0}=0.8$. On the other hand, c,d) illustrate the frequency dynamics for α=0.4. The initial conditions for (c,d) are respectively $p_{0}=0.6$, $q_{0}=0.4$ and $p_{0}=0.4$, $q_{0}=0.6$. Initial conditions for the averaged parts are found from (4).

From Figure 1 it is obvious that the coexistence of different types depends on the initial state of the populations. These effects are represented in **Figure** 2. Here we show the time evolution of cooperators in populations I and II for different initial states. The smooth lines represent the slow‐varying frequencies of cooperators $\bar{p}$ and $\bar{q}$ in populations I and II, respectively. The oscillating curves represent the non‐averaged frequencies of cooperators p and q. The dynamics of these quantities are directly found from (1) and (2). Note that the initial conditions for the slow‐varying and non‐averaged frequencies are not the same, as follows from (5) (see Supporting Information for further details).

Here again we discuss the two cases α=0 and α≠0. In Figure 2a,b we represent the behavior of averaged and non‐averaged frequencies for the α=0 case. The same phenomena for nonzero α are illustrated in Figure 2c,d. In both cases the other nonlinear payoffs β and γ are the same as in Figure 1. The oscillating parts ${\tilde{a}}_{ij}$ and ${\tilde{b}}_{ij}$ are chosen so as to satisfy the conditions (4) and the given values of β and γ.

As seen from Figure 2b,d, both the averaged $\left(\bar{p},\bar{q}\right)$ and non‐averaged (p,q) frequencies go to extinction for the considered initial values. Thus, although it is possible for environmental variations to create a new stable rest point, these variations do not ensure the stable coexistence of different types for all initial conditions.

**Figure 3:**
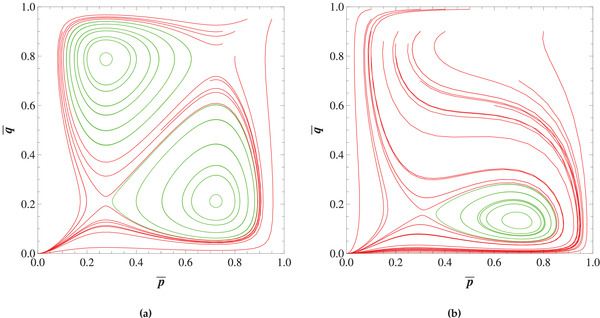
The trajectories of averaged frequencies of cooperators $\left(\bar{p},\bar{q}\right)$ in populations I and II, respectively. The lines represent the behavior of frequencies for different initial conditions. The red lines represent the situation in which the coexistence of different types is impossible. The green lines represent the situations in which cooperators can coexist with defectors. The simulations have been performed for t=10000. a,b) The parameters are chosen to be the same as in Figure 1a,b, respectively.

In **Figure** 3 the behavior of averaged frequencies for different initial conditions is presented. It can be seen that, for given environmental parameters, the coexistence of different types depends on the initial conditions. Note also that high levels of cooperators in both populations in the initial state do not ensure the continued coexistence of cooperators and defectors for all time.

In Figure 3a the two areas in which the coexistence of different types is possible are separated by the trajectories which end up at the mutual defection vertex. The possible states where stable coexistence is possible is narrowed in the α≠0 (Figure 3b) case as compared to the α=0 case (Figure 3a).

## 4 Discussion

In this work, we discuss the problem of cooperation between different populations in a periodically varying environment. To this end, we have focused on the prisoner's dilemma game since it is a game for which mutual defection, an outcome that yields sub‐optimal payoffs for both agents, is the only stable rest point in a fixed environment.

The environmental variations are taken into account as periodic oscillations in the pairwise interaction coefficients, that is, the payoff matrix of the game. These variations occur on the fast timescale. This implies that during the selection process the pairwise interaction coefficients oscillate sufficiently many times, so that we can use the averaging of these oscillations.

The oscillating parts of the pairwise competition coefficients introduce new non‐linear terms in the population dynamics. It has been shown that these new terms will induce nontrivial consequences on the population dynamics if the oscillating parts of the pairwise competition coefficients vary in different phases. Due to these nonlinear terms, the population dynamics can drastically differ from the dynamics in a fixed environment. Indeed, if these terms satisfy certain conditions, then cooperators may coexist with defectors in both populations.

It is noteworthy to emphasize that new rest points arise in the population dynamics in pairs, and that these pairs always include a stable (center) and an unstable (saddle) rest point. We have shown that the appearance of asymptotically stable rest points is impossible. The stable cycles arise in the neighborhood of stable rest points (centers). In these domains, the behavior of the frequencies of cooperators and defectors is similar to the dynamics of some prey‐predator models.

As can be seen in Figure 3a, it is possible to reduce the basin of attraction for the mutual defection point significantly, with much of the phase space replaced by stable cycles, during which the frequency of cooperators in each population roughly follow each other: increasing as the frequency of cooperators in the other population increases, making cooperation more favorable, but decreasing when the frequency of cooperators passes a certain threshold, increasing the temptation to defect (see Figure 2). These results suggest that environmental variations can be a causal mechanism for the emergence of cooperation between different agents, even for games for which standard analyses—assuming a fixed or time‐averaged environment—indicate that mutual defection is the only possible outcome.

We now discuss some possible extensions to this work. The Prisoner's Dilemma game was chosen in this paper because the emergence of cooperation in a game for which mutual defection seems the only possible outcome is an interesting phenomenon for us to investigate. Other games for which the outcome is a foregone conclusion in a fixed environment could be studied to see if—and when—environmental variation can induce new outcomes (stable rest points).

Finally, it is hoped that this paper not only sparks interest in the mechanisms behind the emergence of cooperation (or other interesting outcomes), but also that it promotes further study into the effects of fast‐scale environmental variations on long‐term evolutionary dynamics: as our results show, using the time‐averaged environment is often not sufficient for studying evolutionary dynamics, and the nonlinear effects caused by variations on a fast timescale can produce new, novel and interesting dynamics and outcomes.

## 5 Experimental Section

It is known that the system (1)‐(2) is a Hamiltonian system in the interior of the simplex $S^{1}\times S^{1}$.^[^ ^20^, ^64^ ^]^ Indeed, the dynamical system can be written in the following form:

(10) $$\begin{array}{ccc} & & \frac{dp}{dt}=B\left(p,q\right)\frac{\partial H(p,q)}{\partial q},\;\;\frac{dq}{dt}=-B\left(p,q\right)\frac{\partial H(p,q)}{\partial p}\\ & & H\left(p,q\right)=a_{12}\ln q+a_{21}\ln\left(1-q\right)-b_{21}\ln p-b_{12}\ln\left(1-p\right) \end{array}$$

Here B(p,q)=pq(1−p)(1−q) is an always positive function in the interior of the simplex, so it can be treated as a change of velocity which does not change the dynamical properties; in other words, $\frac{1}{B(p,q)}$ is an integrating factor of the dynamical system (1)‐(2). The dynamical system (1)‐(2) is a volume preserving system, as can be seen by taking the sum of anti‐diagonal elements of the Hessian matrix of the Hamiltonian H(p,q) (see Supporting Information for further details). From this it follows that the existence of asymptotically stable rest points (rest points for which all the eigenvalues of the Jacobian matrix have negative real parts) in the interior of the simplex is impossible. The value of the Hamiltonian function remains constant on any trajectory of the system (1)‐(2) which is in the interior of the simplex $S^{1}\times S^{1}$. Therefore, the Hamiltonian nature of the two‐population replicator dynamics does not contradict the fact that for the prisoner's dilemma game all the interior trajectories converge to the mutual defection vertex (since the Hamiltonian function H(p,q) is not bounded on the boundary of the simplex $S^{1}\times S^{1}$).

In the presence of the periodically varying environment the Hamiltonian nature of the two‐population replicator dynamics is preserved. Indeed, the dynamical system (7)‐(8) is a Hamiltonian system in the interior of the simplex $S^{1}\times S^{1}$ with the same change of velocity $B\left(\bar{p},\bar{q}\right)=\bar{pq}\left(1-\bar{p}\right)\left(1-\bar{q}\right)$, and following Hamiltonian function:

(11) $$H\left(\bar{p},\bar{q}\right)=a\ln\frac{\bar{q}}{1-\bar{q}}-b\ln\frac{\bar{p}}{1-\bar{p}}-\left(\beta \bar{q}-\gamma \bar{p}\right)+\alpha \bar{p}\bar{q}$$

It is obvious that in the absence of environmental variations or in the case where all payoffs oscillate by the same function, that is, α=β=γ=0, the Hamiltonian function for the fixed environment case (10) is recovered. Thus, in this case the dynamical properties are not changed: dynamics of the slow‐varying frequencies are still volume preserving, and hence the existence of asymptotically stable rest points is still impossible.

We can verify this result by considering the Jacobian in the possible rest point in the interior of the $S^{1}\times S^{1}$ simplex. The interior rest points, if they exist, will be defined from the following equations:

(12) $$a-\bar{q}\left(1-\bar{q}\right)\left(\beta -\alpha \bar{p}\right)=0$$

(13) $$b-\bar{p}\left(1-\bar{p}\right)\left(\gamma +\alpha \bar{q}\right)=0$$

Let us denote the possible solution of the above equations by the following pair of frequencies $\left({\bar{p}}^{*},{\bar{q}}^{*}\right)$. The Jacobian matrix in the possible interior rest point is given by

(14) $$\begin{array}{c} J\left({\bar{p}}^{*},{\bar{q}}^{*}\right)=B\left({\bar{p}}^{*},{\bar{q}}^{*}\right)\left[\begin{array}{cc} \alpha & \frac{a(2{\bar{q}}^{*}-1)}{{{\bar{q}}^{*}}^{2}{\left(1-{\bar{q}}^{*}\right)}^{2}}\\ \frac{b(2{\bar{p}}^{*}-1)}{{{\bar{p}}^{*}}^{2}{\left(1-{\bar{p}}^{*}\right)}^{2}} & -\alpha \end{array}\right] \end{array}$$

It is seen that the Jacobian is traceless. Hence, the possible rest points are either centers (with a pair of complex conjugate eigenvalues) or saddles (with one positive and one negative eigenvalue).^[^ ^65^ ^]^ Thus, in contrast to the prisoner's dilemma game in fixed environment case, in the varying environment it is possible that different types can coexist simultaneously. Note that this same result will be obtained if we use the Hessian matrix of the Hamiltonian function at the same rest point instead of the Jacobian matrix.

Let us discuss the conditions which are necessary for the appearance of the new rest points in the dynamics of slow‐varying frequencies. We start from the particular case. Assume that α=0, while β≠γ≠0. These conditions are not mutually excluded as it follows from the definition (6). It is seen from the system (12)‐(13) that in the discussed case the interior rest points are defined from the system of two quadratic equations. The solution of these equations are

(15) $${{\bar{p}}^{*}}_{1,2}=\frac{1}{2}\left(1\pm \sqrt{1-\frac{4b}{\gamma }}\right)$$

(16) $${{\bar{q}}^{*}}_{1,2}=\frac{1}{2}\left(1\pm \sqrt{1-\frac{4a}{\beta }}\right)$$

Now it follows that there are two possibilities: either there will not be any non‐degenerate rest point in the interior of the simplex, or there will be four non‐degenerate rest points. Interior rest points will occur if the conditions γ≤4b and β≤4a are simultaneously satisfied (here we have taken into account that a,b<0), and the set of interior rest points created will exhibit point symmetry about the point $\left(\frac{1}{2},\frac{1}{2}\right)$. In particular, if the inequalities in both conditions are strictly satisfied (i.e., γ<4b and β<4a), then four interior non‐degenerate rest points are created.^[^ ^66^ ^]^

These conditions induce necessary non‐trivial bounds on the environmental variations in order for new rest points to occur. Indeed, it follows that the non‐linear payoffs β and γ—which are induced by environmental variations and defined by the overlaps of the oscillating parts of pairwise coefficients—have to be much larger than the constant parts of pairwise coefficients, which form the prisoner's dilemma game.

As mentioned, it is only possible to get centers or saddles. From (14) it follows that, for the stable rest points (centers) one of the following conditions hold: ${\bar{p}}^{*}>\frac{1}{2},{\bar{q}}^{*}<\frac{1}{2}$ or ${\bar{p}}^{*}<\frac{1}{2},{\bar{q}}^{*}>\frac{1}{2}$. The other two rest points are thus saddle points.^[^ ^67^ ^]^ The new rest points are generated in pairs, and in each pair there is one stable and one unstable rest point.

The further insights about the phase portrait comes from the Hessian matrix of the Hamiltonian function (11) at the possible rest point. This matrix has the following form:

(17) $$\begin{array}{c} Hessian\left[H\left({\bar{p}}^{*},{\bar{q}}^{*}\right)\right]=\left[\begin{array}{cc} \frac{b(1-2{\bar{p}}^{*})}{{{\bar{p}}^{*}}^{2}{\left(1-{\bar{p}}^{*}\right)}^{2}} & \alpha \\ \alpha & \frac{a(2{\bar{q}}^{*}-1)}{{{\bar{q}}^{*}}^{2}{\left(1-{\bar{q}}^{*}\right)}^{2}} \end{array}\right] \end{array}$$

Note that we consider the α=0 case, so from (17) it follows that the rest points ${\bar{p}}^{*}>\frac{1}{2},{\bar{q}}^{*}<\frac{1}{2}$ and ${\bar{p}}^{*}<\frac{1}{2},{\bar{q}}^{*}>\frac{1}{2}$ are respectively the local minimum and maximum of the Hamiltonian function (11).

For the case where α≠0, the expression of the possible rest points does not have as simple a form as that of (15) and (16). Therefore, new non‐degenerate rest points again appear in pairs of stable and unstable points, as is typical for a 1D Hamiltonian system, where the nonlinear resonances occur.

However, in contrast to the particular case, in the general α≠0 case it is possible to obtain only two new non‐degenerate rest points. Stable rest points can be either maxima or minima of the Hamiltonian function depending on the condition ${\bar{p}}^{*}>\frac{1}{2}$, as follows from the Hessian matrix of $H(\bar{p},\bar{q})$. If the rest point is stable, then the determinant of the Hessian is positive and the maximum or minimum condition is governed by the first entity of the Hessian matrix.

Indeed, from (7) and (8) it follows that the new nonlinear terms do not alter the stability of the vertices of $S^{1}\times S^{1}$ and also that it is not possible to make a new rest point on the boundary of the simplex (see Supporting Information for further details). Thus, all the possible rest points will be in the interior of the simplex, and it turns out that we can study the emergence of new rest points with Poincare index theory (see Supporting Information).

It is known that the two‐population asymmetric replicator dynamics (1)‐(2) admits time‐averaging properties.^[^ ^20^ ^]^ If the trajectory (p(t),q(t)) remains in the interior of the simplex $S^{1}\times S^{1}$ during the time evolution of the system, then the time‐average of the trajectory is determined by the interior rest point. Obviously, this property does not hold for the prisoner's dilemma game in the fixed environment, since there is no rest point in the interior of the simplex and all the trajectories go to the mutual defection vertex. However, as we have seen above, in a varying environment new rest points can appear in the interior of the simplex. Thus, for the dynamics (7)‐(8), and in the α=0 case, we get the following relations for a trajectory$\left(\bar{p}\left(t\right),\bar{q}\left(t\right)\right)$ that always remains in the interior of the simplex, for large time T:

(18) $$\frac{1}{T}\int_{0}^{T}\bar{p}\left(t\right)\left(1-\bar{p}\left(t\right)\right)dt=\frac{b}{\gamma }={\bar{p}}^{*}\left(1-{\bar{p}}^{*}\right)$$

(19) $$\frac{1}{T}\int_{0}^{T}\bar{q}\left(t\right)\left(1-\bar{q}\left(t\right)\right)dt=\frac{a}{\beta }={\bar{q}}^{*}\left(1-{\bar{q}}^{*}\right)$$

Thus (18) and (19) give us the time‐averaging property for the slow‐varying frequencies. Depending on the initial conditions of slow‐varying frequencies, the time‐average of $\bar{p}\left(t\right)\left(1-\bar{p}\left(t\right)\right)$ and $\bar{q}\left(t\right)\left(1-\bar{q}\left(t\right)\right)$ will be defined respectively from (15) and (16). Note that when the interior rest point—defined by (15) and (16)—does not exist, we get a contradiction in (18) and (19). In this case our initial assumption that the trajectory $\left(\bar{p}\left(t\right),\bar{q}\left(t\right)\right)$ always remains in the interior of the simplex is not valid: the trajectory must thus end up on the boundary of the simplex.

## Conflict of Interest

The authors declare no conflict of interest.

## Supporting information

Supporting Information
