# Supplementary material for: Cooperate or Not Cooperate in Predictable but Periodically Varying Situations? Cooperation in Fast Oscillating Environment
Source: Adv Sci (Weinh). 2020 Sep 18;7(21):2001995. doi: 10.1002/advs.202001995 (PMC7610311; doi:10.1002/advs.202001995)
Supplement: Supplementary file 1 — Supporting Information [file ADVS-7-2001995-s001.pdf]

© 2020 Wiley-VCH GmbH

ADVANCED SCIENCE
Open Access

Supporting Information

for *Adv. Sci.*, DOI: 10.1002/advs.202001995

Cooperate or Not Cooperate in Predictable but Periodically Varying Situations? Cooperation in Fast Oscillating Environment

*S. G. Babajanyan, Wayne Lin, and Kang Hao Cheong**

Supporting Information

Replicator dynamics

In evolutionary game theory the players are associated with populations, and the strategies with the types (traits, species) within each population[20]. The payoff matrix describes the evolutionary outcome of the pairwise interaction between types from different populations, e.g. for cooperators from each population it is preferable to interact with other cooperators than defectors. We will assume that the populations are well mixed, i.e. the probability that two agents meet is defined by the frequencies of their types in their populations. The growth rate of a type is described by its relative fitness. For the two-population case, the fitness of a given type in one population depends on the frequencies of different types in the other population. Fitness can also be thought of as the expected payoff of a player whose opponent is playing by mixed strategies.

The change of the frequencies of each type in each population is governed by the replicator equation. For the two-population case, the replicator dynamics has the following form:

$$\frac{dp_i}{dt} = p_i(\sum_{l}^{M} a_{il}q_l - \sum_{k,l}^{N,M} p_k a_{kj} q_l), \tag{20}$$

$$\frac{dq_j}{dt} = q_j(\sum_{l}^{N} b_{jl}p_l - \sum_{k,l}^{N,M} q_l b_{kl} p_k), \tag{21}$$

where $p_i,\ i = 1,..,N$ ($q_j, j = 1,..,M$) represents the frequency of type i (j) in the first (second) population, which consist of $N(M)$ different types. The matrix elements a_{il} (b_{jk}) represent the pairwise interaction outcome between the types i (j) from the first(second) population and l (k) from the second (first) population. Success of a given type i is governed by the fitness of that type: this is the first term in the right hand-side of (20) and (21). The frequency of a given type

will increase if the fitness of that type (or expected payoff of the given strategy) is higher than the mean fitness of the population to which it corresponds. Note that the fitness of a given type from one population is defined by the interaction with the other population, i.e. different types from the same population don't interact with each other. Thus, a given type from a population interacts with the members of other population, but the selection is through its own population. Furthermore, it follows from (20) and (21) that if a given type is absent from the population initially, i.e. $p_i = 0$ (or $q_j = 0$), then this type will not occur during the time evolution of the system. It follows from the system of equations (20) and (21) that the sum of the frequencies $\sum_i^N p_i = 1$ ($\sum_j^M q_j = 1$) remains invariant during the time evolution. Thus, the dynamical system (20)-(21) is defined on the $S^{N-1} \times S^{M-1}$ simplex, where $S^{N-1} = \left\{ p \in R^N | p_i \geq 0, \; \sum_i^N p_i = 1 \right\}$ (same for the second population), and this simplex and its faces are invariant. Hereon, we will focus on the two-population, two-type case, i.e. $N = M = 2$.

Two-population, two-type replicator dynamics in fixed environment

We recall here the dynamical equations for the fixed environment case:

$$\frac{dp}{dt} = p(1-p)(a_{12} - (a_{12} + a_{21})q), \tag{22}$$

$$\frac{dq}{dt} = q(1-q)(b_{21} - (b_{21} + b_{12})p), \tag{23}$$

under the conditions

$$a_{12} < 0 < a_{21}, \tag{24}$$

$$b_{21} < 0 < b_{12}. \tag{25}$$

Vertices are the only rest points

We clearly see that any vertex of $S^1 \times S^1$, i.e. any of ($(0,0)$, $(0,1)$, $(1,0)$, or $(1,1)$) is a rest point, since at a vertex we have that $p(1-p) = q(1-q) = 0$.

We next see that the boundary can admit no further rest points: consider a point on the $p = 0$ boundary that is not a corner point. Then we have $\frac{dp}{dt} = 0$, but $\frac{dq}{dt} = q(1-q)b_{21} < 0$, so the point is not a rest point. Points on the other edges of the boundary can be considered similarly, and shown to be not rest points by using the conditions (24) and (25).

Finally, we see that dynamics admits no rest points in the interior of $S^1 \times S^1$, since for all $(p,q) \in S^1 \times S^1$ we have that

$$a_{12} - (a_{12} + a_{21})q = a_{12}(1-q) - a_{21}q < 0 \tag{26}$$

by condition (24), so for all $(p,q) \in (0,1)^2$ we have that $\frac{dp}{dt} < 0$ by the dynamical equation (22).

Thus, the only rest points are the vertices of $S^1 \times S^1$: $(0,0)$, $(0,1)$, $(1,0)$, and $(1,1)$.

(0,0) is the only stable rest point

From the dynamical equations (22) and (23), we can compute the Jacobian matrix

$$J = \begin{pmatrix} \frac{\partial \dot{p}}{\partial p} & \frac{\partial \dot{p}}{\partial q} \\ \frac{\partial \dot{q}}{\partial p} & \frac{\partial \dot{q}}{\partial q} \end{pmatrix} = \begin{pmatrix} (1-2p)(a_{12} - (a_{12}+a_{21})q) & -p(1-p)(a_{12}+a_{21}) \\ -q(1-q)(b_{21}+b_{12}) & (1-2q)(b_{21} - (b_{21}+b_{12})p) \end{pmatrix}, \tag{27}$$

which at a corner point ($p(1-p) = q(1-q) = 0$) becomes

$$J = \begin{pmatrix} (1-2p)(a_{12} - (a_{12}+a_{21})q) & 0 \\ 0 & (1-2q)(b_{21} - (b_{21}+b_{12})p) \end{pmatrix}. \tag{28}$$

We have established in (26) that $a_{12} - (a_{12} + a_{21})q < 0$, and can show similarly that $b_{21} - (b_{21} + b_{12})p < 0$. Hence we have that the signs of the eigenvalues of the Jacobian matrix depend only on the signs of $(1-2p)$, $(1-2q)$, i.e. which quadrant the corner point is in. Thus:

- At $(0,0)$: $1-2p$, $1-2q > 0$ so both eigenvalues are real and negative, and hence we have a stable rest point.
- At $(0,1)$ and $(1,0)$: $1-2p$, $1-2q$ are of opposite sign, and thus the two eigenvalues are real and of opposite sign. Hence we have a saddle point.
- At $(1,1)$: $1-2p$, $1-2q < 0$, so both eigenvalues are real and positive, and hence we have an unstable rest point.

Hessian of Hamiltonian in the interior

It can be verified that

$$H(p,q) = a_{12}\ln q + a_{21}\ln(1-q) - b_{21}\ln p - b_{12}\ln(1-p), \tag{29}$$

is a Hamiltonian for the dynamics (for fixed environment) in the interior of $S^1 \times S^1$. From this, we can compute the first partial derivatives

$$\frac{\partial H}{\partial p} = -\frac{b_{21}}{p} - \frac{b_{12}}{1-p}, \tag{30}$$

$$\frac{\partial H}{\partial q} = \frac{a_{12}}{p} + \frac{a_{21}}{1-q}. \tag{31}$$

We can then compute the elements of the Hessian matrix:

$$\frac{\partial^2 H}{\partial p^2} = \frac{b_{21}}{p^2} - \frac{b_{12}}{(1-p)^2}, \tag{32}$$

$$\frac{\partial^2 H}{\partial q^2} = -\frac{a_{12}}{q^2} + \frac{a_{21}}{(1-q)^2}, \tag{33}$$

$$\frac{\partial^2 H}{\partial p \partial q} = \frac{\partial^2 H}{\partial q \partial p} = 0. \tag{34}$$

This gives us the Hessian matrix,

$$Hessian(H) = \begin{pmatrix} \frac{\partial^2 H}{\partial p^2} & \frac{\partial^2 H}{\partial p \partial q} \\ \frac{\partial^2 H}{\partial q \partial p} & \frac{\partial^2 H}{\partial q^2} \end{pmatrix} = \begin{pmatrix} \frac{b_{21}}{p^2} - \frac{b_{12}}{(1-p)^2} & 0 \\ 0 & -\frac{a_{12}}{q^2} + \frac{a_{21}}{(1-q)^2} \end{pmatrix}. \tag{35}$$

Derivation of dynamical equations for slow-varying terms

In the presence of a varying environment that is incorporated into the system as oscillating payoffs, replicator dynamics gives us the equations

$$\frac{dp}{dt} = p(1-p)\left(a_{12}(\tau) - \big(a_{12}(\tau) + a_{21}(\tau)\big)\, q\right), \tag{36}$$

$$\frac{dq}{dt} = q(1-q)\left(b_{21}(\tau) - \big(b_{21}(\tau) + b_{12}(\tau)\big)\, p\right). \tag{37}$$

(Note that we have assumed $a_{11}(\tau) = a_{22}(\tau) = b_{11}(\tau) = b_{22}(\tau) = 0$. We can do this because, as per the discussion for the fixed environment case, we can at every moment in time add a constant to the columns of the matrix a_{ij} and the rows of the matrix b_{ij} so that the diagonal terms of the payoff matrix are 0 without changing the dynamics of the system.)

Let us now, for the sake of convenience, define the functions

$$\begin{aligned} A(\tau) &\equiv a_{12}(\tau), \\ B(\tau) &\equiv a_{12}(\tau) + a_{21}(\tau), \\ C(\tau) &\equiv b_{21}(\tau), \\ D(\tau) &\equiv b_{21}(\tau) + b_{12}(\tau). \end{aligned} \tag{38}$$

Furthermore we define the function

$$G\big(A, B, p, q\big) \equiv p(1-p)\big(A - Bq\big), \tag{39}$$

so that we can rewrite (36) and (37) as

$$\frac{dp}{dt} = G\big(A(\tau), B(\tau), p(t), q(t)\big), \tag{40}$$

$$\frac{dq}{dt} = G\big(C(\tau), D(\tau), q(t), p(t)\big). \tag{41}$$

Our aim now is to split the components of equation (40) (and similarly, (41)) into a slow-varying part and a fast-varying part. For the left-hand side of (40), we do this by taking the total derivative of $\epsilon\,\big(\bar{p}(t),\,\bar{q}(t),\,\tau\big)$ with respect to t, from which we obtain

$$\frac{dp}{dt} = \frac{d\bar{p}}{dt} + \frac{\partial \epsilon}{\partial \bar{p}}\frac{d\bar{p}}{dt} + \frac{\partial \epsilon}{\partial \bar{q}}\frac{d\bar{q}}{dt} + \omega \partial_\tau \epsilon, \tag{42}$$

where $\partial_\tau \epsilon$ is the partial derivative of $\epsilon(\bar{p},\,\bar{q},\,\tau)$ taken with respect to τ.

For the right-hand side of equation (40), we simply take the Taylor expansion of G about $(\bar{p}, \bar{q})$ to get

$$G\big(A(\tau), B(\tau), p(t), q(t)\big) = \big(1 + \epsilon\partial_{\bar{p}} + \eta\partial_{\bar{q}}\big)\Big[G\big(\overline{A}, \overline{B}, \bar{p}, \bar{q}\big) + G\big(\widetilde{A}(\tau), \widetilde{B}(\tau), \bar{p}, \bar{q}\big)\Big] + O(\frac{1}{\omega^2}), \tag{43}$$

where

$$\begin{aligned} \overline{A} &\equiv \bar{a}_{12}, \\ \widetilde{A}(\tau) &\equiv \widetilde{a}_{12}(\tau) + \widetilde{a}_{21}(\tau). \end{aligned} \tag{44}$$

Now, equating expressions (42) and (43) and taking the fast-varying, $O(1)$ terms, we get

$$\omega \partial_\tau \epsilon = G\big(\widetilde{A}, \widetilde{B}, \bar{p}, \bar{q}\big) + O(\frac{1}{\omega}), \tag{45}$$

which we can integrate to get

$$\epsilon = \frac{1}{\omega} G\big(\widehat{A}, \widehat{B}, \bar{p}, \bar{q}\big) + O(\frac{1}{\omega^2}), \tag{46}$$

where $\widehat{A}(\tau)$, $\widehat{B}(\tau)$ are primitives of $\widetilde{A}(\tau)$, $\widetilde{B}(\tau)$ respectively, i.e.

$$\partial_\tau \widehat{A}(\tau) = \widetilde{A}(\tau), \qquad \int_0^{2\pi} \hat{A}(\tau)\frac{d\tau}{2\pi} = 0. \tag{47}$$

Similarly we get

$$\eta = \frac{1}{\omega} G(\widehat{C}, \widehat{D}, \bar{q}, \bar{p}) + O(\frac{1}{\omega^2}). \tag{48}$$

We can then substitute the expressions (46) and (48) into equation (43) to get

$$\frac{dp}{dt} = G(\overline{A}, \overline{B}, \bar{p}, \bar{q}) + G(\widetilde{A}, \widetilde{B}, \bar{p}, \bar{q}) + \frac{1}{\omega} G(\widehat{A}, \widehat{B}, \bar{p}, \bar{q}) \partial_{\bar{p}} G(A, B, \bar{p}, \bar{q}) + \frac{1}{\omega} G(\widehat{C}, \widehat{D}, \bar{q}, \bar{p}) \partial_{\bar{q}} G(A, B, \bar{p}, \bar{q}) + O(\frac{1}{\omega^2}). \tag{49}$$

Now take the time-average over a period to get the slow-time variation:

$$\frac{d\bar{p}}{dt} = G(\overline{A}, \overline{B}, \bar{p}, \bar{q}) + \frac{1}{\omega} \overline{G(\widehat{A}, \widehat{B}, \bar{p}, \bar{q}) \partial_{\bar{p}} G(A, B, \bar{p}, \bar{q})} + \frac{1}{\omega} \overline{G(\widehat{C}, \widehat{D}, \bar{q}, \bar{p}) \partial_{\bar{q}} G(A, B, \bar{p}, \bar{q})} + O(\frac{1}{\omega^2}). \tag{50}$$

But we have

$$\begin{aligned}
\overline{G(\widehat{A}, \widehat{B}, \bar{p}, \bar{q}) \partial_{\bar{p}} G(A, B, \bar{p}, \bar{q})} &= \overline{G(\widehat{A}, \widehat{B}, \bar{p}, \bar{q}) \partial_{\bar{p}} G(\overline{A}, \overline{B}, \bar{p}, \bar{q})} + \overline{G(\widehat{A}, \widehat{B}, \bar{p}, \bar{q}) \partial_{\bar{p}} G(\widetilde{A}, \widetilde{B}, \bar{p}, \bar{q})} \\
&= O\left(\frac{1}{\omega}\right),
\end{aligned} \tag{51}$$

since from (46) we have that

$$\begin{aligned}
\overline{G(\widehat{A}, \widehat{B}, \bar{p}, \bar{q}) \partial_{\bar{p}} G(\overline{A}, \overline{B}, \bar{p}, \bar{q})} &= \overline{G(\widehat{A}, \widehat{B}, \bar{p}, \bar{q})} \cdot \partial_{\bar{p}} G(\overline{A}, \overline{B}, \bar{p}, \bar{q}) \\
&= \overline{\omega\epsilon + O\left(\frac{1}{\omega}\right)} \cdot \partial_{\bar{p}} G(\overline{A}, \overline{B}, \bar{p}, \bar{q}) \\
&= \omega\bar{\epsilon} \cdot \partial_{\bar{p}} G(\overline{A}, \overline{B}, \bar{p}, \bar{q}) + O\left(\frac{1}{\omega}\right) \\
&= O\left(\frac{1}{\omega}\right),
\end{aligned} \tag{52}$$

and

$$\begin{aligned}
\overline{G(\widehat{A}, \widehat{B}, \bar{p}, \bar{q}) \partial_{\bar{p}} G(\widetilde{A}, \widetilde{B}, \bar{p}, \bar{q})} &= \overline{\left[\bar{p}(1-\bar{p})(\widehat{A} - \widehat{B}\bar{q})\right] \left[(1-2\bar{p})(\widetilde{A} - \widetilde{B}\bar{q})\right]} \\
&= \bar{p}(1-\bar{p})(1-2\bar{p}) \int_0^{2\pi} (\widehat{A} - \widehat{B}\bar{q})(\widetilde{A} - \widetilde{B}\bar{q}) \frac{d\tau}{2\pi} \\
&= \frac{\bar{p}(1-\bar{p})(1-2\bar{p})}{2\pi} \int_0^{2\pi} (\widehat{A} - \widehat{B}\bar{q}) d(\widehat{A} - \widehat{B}\bar{q}) \\
&= \frac{\bar{p}(1-\bar{p})(1-2\bar{p})}{2\pi} \cdot \left[\widehat{A} - \widehat{B}\bar{q}\right]^2 \Big|_{\tau=0}^{2\pi} \\
&= 0.
\end{aligned} \tag{53}$$

Thus (50) becomes

$$
\begin{aligned}
\frac{d\bar{p}}{dt} &= G\big(\overline{A},\overline{B},\bar{p},\bar{q}\big) + \frac{1}{\omega}\overline{G\big(\widehat{C},\widehat{D},\bar{q},\bar{p}\big)\partial_{\bar{q}}G\big(A,B,\bar{p},\bar{q}\big)} + O\left(\frac{1}{\omega^2}\right) \\
&= G\big(\overline{A},\overline{B},\bar{p},\bar{q}\big) - \frac{1}{\omega}\bar{p}\bar{q}(1-\bar{p})(1-\bar{q})\overline{\big(\widehat{C}-\widehat{D}\bar{p}\big)\widetilde{B}} + O\left(\frac{1}{\omega^2}\right),
\end{aligned}
\tag{54}
$$

and similarly we get that the slow-time variation of q is

$$
\frac{d\bar{q}}{dt} = G\big(\overline{C},\overline{D},\bar{q},\bar{p}\big) - \frac{1}{\omega}\bar{p}\bar{q}(1-\bar{p})(1-\bar{q})\overline{\big(\widehat{A}-\widehat{B}\bar{q}\big)\widetilde{D}} + O\left(\frac{1}{\omega^2}\right).
\tag{55}
$$

Now we observe that

$$
\begin{aligned}
\overline{\widehat{D}\widetilde{B}} &= \int_0^{2\pi} \widehat{D}\widetilde{B}\frac{d\tau}{2\pi} \\
&= \frac{1}{2\pi}\int_0^{2\pi} \widehat{D}d\widehat{B} \\
&= \frac{1}{2\pi}\left[\widehat{D}\widehat{B}\Big|_{\tau=0}^{2\pi} - \int_0^{2\pi} \widehat{B}d\widehat{D}\right] \\
&= -\overline{\widehat{B}\widetilde{D}},
\end{aligned}
\tag{56}
$$

so by defining the parameters

$$
\begin{aligned}
\alpha &\equiv \frac{1}{\omega}\overline{\widehat{D}\widetilde{B}} = \frac{1}{\omega}\overline{(\widehat{b}_{21}+\widehat{b}_{12})(\widetilde{a}_{12}+\widetilde{a}_{21})}, \\
\beta &\equiv \frac{1}{\omega}\overline{\widehat{C}\widetilde{B}} = \frac{1}{\omega}\overline{\widehat{b}_{21}(\widetilde{a}_{12}+\widetilde{a}_{21})}, \\
\gamma &\equiv \frac{1}{\omega}\overline{\widehat{A}\widetilde{D}} = \frac{1}{\omega}\overline{\widehat{a}_{12}(\widetilde{b}_{21}+\widetilde{b}_{12})}
\end{aligned}
\tag{57}
$$

and dropping the $O\left(\frac{1}{\omega}\right)$ terms, we get the dynamical equations

$$
\frac{d\bar{p}}{dt} = G\big(\overline{A},\overline{B},\bar{p},\bar{q}\big) - \bar{p}(1-\bar{p})\bar{q}(1-\bar{q})(\beta-\alpha\bar{p}),
\tag{58}
$$

$$
\frac{d\bar{q}}{dt} = G\big(\overline{C},\overline{D},\bar{q},\bar{p}\big) - \bar{q}(1-\bar{q})\bar{p}(1-\bar{p})(\gamma+\alpha\bar{q}),
\tag{59}
$$

which written out fully give

$$
\frac{d\bar{p}}{dt} = \bar{p}(1-\bar{p})\big[(\overline{A}-\overline{B}\bar{q}) - \bar{q}(1-\bar{q})(\beta-\alpha\bar{p})\big],
\tag{60}
$$

$$\frac{d\bar{q}}{dt} = \bar{q}(1-\bar{q})\big[(\overline{C} - \overline{D}\bar{p}) - \bar{p}(1-\bar{p})(\gamma + \alpha\bar{q})\big]. \tag{61}$$

Now we input the further simplification that $\bar{a}_{12} = -\bar{a}_{21} = a < 0$ and $\bar{b}_{21} = -\bar{b}_{12} = b < 0$, which translate to

$$\begin{aligned} \overline{A} &= a, \\ \overline{C} &= b, \\ \overline{B} &= \overline{D} = 0. \end{aligned} \tag{62}$$

This gives us the final form of the dynamical equations,

$$\frac{d\bar{p}}{dt} = \bar{p}(1-\bar{p})\big[a - \bar{q}(1-\bar{q})(\beta - \alpha\bar{p})\big], \tag{63}$$

$$\frac{d\bar{q}}{dt} = \bar{q}(1-\bar{q})\big[b - \bar{p}(1-\bar{p})(\gamma + \alpha\bar{q})\big]. \tag{64}$$

Two-population replicator dynamics in fast periodically varying environment

Vertices remain rest points, no new rest points created on the boundary

From the dynamical equations (63) and (64), it is easy to see that the vertices of $S^1 \times S^1$ remain rest points.

To see that no new rest points are created on the boundary, consider a point on the $p = 0$ boundary that is not a vertex. By (64) we have that $\frac{dq}{dt} = \bar{q}(1-\bar{q})b < 0$, so this point is not a rest point. Similarly, we can show that the other edges admit no rest points apart from the vertices of $S^1 \times S^1$.

Stability of the corner rest points unchanged by environmental oscillation

Let us compare the case with environmental variation to the case with fixed environment. To do this, substitute the parameters for the fixed environment ($\widetilde{A}(\tau) = \widetilde{B}(\tau) = \widetilde{C}(\tau) = \widetilde{D}(\tau) = 0$) into

the equations (40), (41). This gives the dynamical equations $\frac{dp}{dt} = G(\overline{A}, \overline{B}, p, q)$, $\frac{dq}{dt} = G(\overline{C}, \overline{D}, q, p)$. We note that the dynamical equations for the oscillating environment, written in the form of (58) and (59), are very similar to these equations for the fixed environment, just modified by a term that is very small at the corner points. Thus, we should expect the dynamics near the corner points to be the same as that in the fixed environment. (Indeed, because the extra term is "doubly zero" at each corner point, it will not contribute anything to the Jacobian, i.e. the Jacobian at the corner points would be the same with or without environmental oscillation.)

Nevertheless, we present below rigorous computation of the Jacobian to find the stability of the corner points:

From the dynamical equations (63) and (64), and noting that at the corner points we have

$$\bar{p}(1-\bar{p}) = \bar{q}(1-\bar{q}) = 0, \tag{65}$$

we can compute the entries of the Jacobian matrix at the corner points:

$$\begin{aligned} \frac{\partial \dot{\bar{p}}}{\partial \bar{p}} &= \frac{\partial}{\partial \bar{p}}\Big[\bar{p}(1-\bar{p})\Big] \cdot \big[a - \bar{q}(1-\bar{q})(\beta - \alpha\bar{p})\big] + \bar{p}(1-\bar{p}) \cdot \frac{\partial}{\partial \bar{p}}\Big[a - \bar{q}(1-\bar{q})(\beta - \alpha\bar{p})\Big] \\ &= (1-2\bar{p}) \cdot \big[a - \bar{q}(1-\bar{q})(\beta - \alpha\bar{p})\big] + 0 \\ &= (1-2\bar{p})a, \end{aligned} \tag{66}$$

$$\begin{aligned} \frac{\partial \dot{\bar{p}}}{\partial \bar{q}} &= \bar{p}(1-\bar{p}) \cdot \frac{\partial}{\partial \bar{q}}\Big[a - \bar{q}(1-\bar{q})(\beta - \alpha\bar{p})\Big] \\ &= 0, \end{aligned} \tag{67}$$

and similarly

$$\begin{aligned} \frac{\partial \dot{\bar{q}}}{\partial \bar{p}} &= 0, \\ \frac{\partial \dot{\bar{p}}}{\partial \bar{p}} &= (1-2\bar{q})b. \end{aligned} \tag{68}$$

(Here, $\dot{\bar{p}}$ denotes $\frac{d\bar{p}}{dt}$.)

This gives us the Jacobian matrix at the corner points,

$$J = \begin{pmatrix} \frac{\partial \dot{p}}{\partial p} & \frac{\partial \dot{p}}{\partial q} \\ \frac{\partial \dot{q}}{\partial p} & \frac{\partial \dot{q}}{\partial q} \end{pmatrix} = \begin{pmatrix} (1-2p)a & 0 \\ 0 & (1-2q)b \end{pmatrix}. \tag{69}$$

Since we have that $a, b < 0$, we have that the first and second eigenvalues of J are negative iff $1 - 2p > 0$ and $1 - 2q > 0$ respectively. Thus:

- At $(0, 0)$: both $1 - 2p$ and $1 - 2q$ are positive, so both eigenvalues are negative and we have a stable rest point.
- At $(0, 1)$ and $(1, 0)$: $1 - 2p$ and $1 - 2q$ are of opposite sign, so the eigenvalues are of opposite sign and we have saddle points.
- At $(1, 1)$: both $1 - 2p$ and $1 - 2q$ are negative, so both eigenvalues are positive and we have a unstable node.

Thus we see that the stability of the corner rest points are unchanged by the oscillations in the environment.

Computation of Jacobian at possible interior rest point

At an interior rest point $(\bar{p}^*, \bar{q}^*)$, we have from $\dot{\bar{p}} = \dot{\bar{q}} = 0$ that

$$a - \bar{q}^*(1 - \bar{q}^*)(\beta - \alpha\bar{p}^*) = b - \bar{p}^*(1 - \bar{p}^*)(\gamma + \alpha\bar{q}^*) = 0. \tag{70}$$

Using this, we can compute the elements of the Jacobian matrix:

$$\left.\frac{\partial\dot{\bar{p}}}{\partial\bar{p}}\right|_{(\bar{p}^*,\bar{q}^*)} = \left.\frac{\partial}{\partial\bar{p}}\Big[\bar{p}(1-\bar{p})\Big]\right|_{(\bar{p}^*,\bar{q}^*)} \cdot \Big[a - \bar{p}^*(1-\bar{q}^*)(\beta-\alpha\bar{p}^*)\Big] + \bar{p}^*(1-\bar{q}^*)\cdot\left.\frac{\partial}{\partial\bar{p}}\Big[a - \bar{q}(1-\bar{q})(\beta-\alpha\bar{p})\Big]\right|_{(\bar{p}^*,\bar{q}^*)}$$

$$= 0 + \bar{p}^*(1-\bar{p}^*)\left[\bar{q}^*(1-\bar{q}^*)\alpha\right]$$

$$= B(\bar{p}^*,\bar{q}^*)\alpha, \tag{71}$$

$$\begin{aligned}\left.\frac{\partial\dot{\bar{p}}}{\partial\bar{q}}\right|_{(\bar{p}^*,\bar{q}^*)} &= \bar{p}^*(1-\bar{p}^*)\cdot\left.\frac{\partial}{\partial\bar{q}}\Big[[a - \bar{q}(1-\bar{q})(\beta-\alpha\bar{p})]\Big]\right|_{(\bar{p}^*,\bar{q}^*)} \\ &= \bar{p}^*(1-\bar{p}^*)(2\bar{q}^*-1)(\beta-\alpha\bar{p}^*) \\ &= \bar{p}^*(1-\bar{p}^*)(2\bar{q}^*-1)\cdot\frac{a}{\bar{q}^*(1-\bar{q}^*)} \\ &= B(\bar{p}^*,\bar{q}^*)\cdot\frac{a(2\bar{q}^*-1)}{\bar{q}^{*2}(1-\bar{q}^*)^2},\end{aligned} \tag{72}$$

and similarly

$$\left.\frac{\partial\dot{\bar{q}}}{\partial\bar{p}}\right|_{(\bar{p}^*,\bar{q}^*)} = B(\bar{p}^*,\bar{q}^*)\cdot\frac{b(2\bar{p}^*-1)}{\bar{p}^{*2}(1-\bar{p}^*)^2}, \tag{73}$$

$$\left.\frac{\partial\dot{\bar{q}}}{\partial\bar{q}}\right|_{(\bar{p}^*,\bar{q}^*)} = -B(\bar{p}^*,\bar{q}^*)\alpha. \tag{74}$$

(Note the minus sign in $\frac{\partial\dot{\bar{q}}}{\partial\bar{q}}$ because the sign of the α term in the expression for $\dot{\bar{q}}$ is the opposite of the sign of the α term in the expression for $\dot{\bar{p}}$.)

This gives us the Jacobian at the possible interior rest point,

$$J(\bar{p}^*,\bar{q}^*) = \begin{pmatrix} \frac{\partial\dot{p}}{\partial p} & \frac{\partial\dot{p}}{\partial q} \\ \frac{\partial\dot{q}}{\partial p} & \frac{\partial\dot{q}}{\partial q} \end{pmatrix} = B(\bar{p}^*,\bar{q}^*)\begin{pmatrix} \alpha & \frac{a(2\bar{q}^*-1)}{\bar{q}^{*2}(1-\bar{q}^*)^2} \\ \frac{b(2\bar{p}^*-1)}{\bar{p}^{*2}(1-\bar{p}^*)^2} & -\alpha \end{pmatrix}. \tag{75}$$

We see that the Jacobian is traceless, so any interior rest point must be either a center or a saddle.

Hessian matrix of Hamiltonian in interior

From the Hamiltonian

$$H(p,q) = a\ln\frac{\bar{q}}{1-\bar{q}} - b\ln\frac{\bar{p}}{1-\bar{p}} - (\beta\bar{q} - \gamma\bar{p}) + \alpha\bar{p}\bar{q}, \tag{76}$$

we can compute the first partial derivatives

$$\frac{\partial H}{\partial p} = -\frac{b}{\bar{p}(1-\bar{p})} + \gamma + \alpha\bar{q}, \tag{77}$$

$$\frac{\partial H}{\partial q} = \frac{a}{\bar{q}(1-\bar{q})} - \beta + \alpha\bar{p}. \tag{78}$$

We can then compute the elements of the Hessian matrix:

$$\frac{\partial^2 H}{\partial p^2} = \frac{b}{\bar{p}^2(1-\bar{p})^2}\cdot(1-2\bar{p}), \tag{79}$$

$$\frac{\partial^2 H}{\partial p\partial q} = \frac{\partial^2 H}{\partial q\partial p} = \alpha, \tag{80}$$

$$\frac{\partial^2 H}{\partial q^2} = -\frac{a}{\bar{q}^2(1-\bar{q})^2}\cdot(1-2\bar{q}) = \frac{a(2\bar{q}-1)}{\bar{q}^2(1-\bar{q})^2}. \tag{81}$$

This gives us the Hessian matrix,

$$Hessian(H) = \begin{pmatrix} \frac{\partial^2 H}{\partial p^2} & \frac{\partial^2 H}{\partial p\partial q} \\ \frac{\partial^2 H}{\partial q\partial p} & \frac{\partial^2 H}{\partial q^2} \end{pmatrix} = \begin{pmatrix} \frac{b(1-2\bar{p})}{\bar{p}^2(1-\bar{p})^2} & \alpha \\ \alpha & \frac{a(2\bar{q}-1)}{\bar{q}^2(1-\bar{q})^2} \end{pmatrix}. \tag{82}$$

Thus, for the $\alpha = 0$ case, the anti-diagonal components vanish and we have that:

- for the interior rest point $(\bar{p}^*, \bar{q}^*)$ with $\bar{p}^* > \frac{1}{2}$, $\bar{q}^* < \frac{1}{2}$, the Hessian matrix is positive definite and so the rest point is a local minimum of the Hamiltonian function, and
- for the interior rest point $(\bar{p}^*, \bar{q}^*)$ with $\bar{p}^* < \frac{1}{2}$, $\bar{q}^* > \frac{1}{2}$, the Hessian matrix is negative definite and so the rest point is a local maximum of the Hamiltonian function.

New rest points emerge in pairs due to Poincare index theory

We now recall the Poincare index of rest points and curves[65]. For the two-dimensional case, the index of a rest point is given by the sign of the determinant of the Jacobian matrix $J(\overline{p}^*, \overline{q}^*)$ at that point:

$$\mathrm{Ind}[(\overline{\mathrm{p}}^*, \overline{\mathrm{q}}^*)] = \mathrm{sgn}\ \det[\mathrm{J}(\overline{\mathrm{p}}^*, \overline{\mathrm{q}}^*)]. \tag{83}$$

Here $(\overline{p}^*, \overline{q}^*)$ is a possible interior rest point of the system (15)-(16). Note that the index of a center is $+1$ and that of a saddle is -1.

The index of a closed curve is equal to the sum of the indices of the non-degenerate rest points enclosed by that curve :

$$\mathrm{Ind}[\mathrm{C}] = \sum_{\mathrm{k}} \mathrm{Ind}[\pi_{\mathrm{k}}] \tag{84}$$

where $\pi_k = (\overline{p}^*, \overline{q}^*)_k$ denote the possible enclosed rest points. We know that the dynamical system (15)-(16) does not admit any rest point in the interior of the simplex when environmental variations are absent, i.e $\alpha = \beta = \gamma = 0$. Thus, in this case the index of a curve C drawn close to the boundary would be equal to 0.

This would mean that, if environmental variations induce new rest points all of which are in the interior of the curve C, then the number of these new rest points has to be even, and the numbers of stable and unstable rest points have to be equal. This is due to the fact that as α, β, and γ are varied the index must remain constant, since it is a continuous function of the parameters that takes only integer values.

Time-averaging properties for $\alpha = 0$ case

Since $\alpha = 0$, the expression for $\dot{\bar{q}}$ becomes

$$\dot{\bar{q}} = \bar{q}(1-\bar{q})(b - \gamma\bar{p}(1-\bar{p})). \tag{85}$$

Thus we have that, in the interior of the simplex,

$$b - \gamma\bar{p}(1-\bar{p}) = \frac{1}{\bar{q}(1-\bar{q})}\dot{\bar{q}} = \frac{d}{dt}\ln\frac{\bar{q}}{1-\bar{q}}, \tag{86}$$

and so

$$\bar{p}(1-\bar{p}) = \frac{b}{\gamma} - \frac{1}{\gamma}\frac{d}{dt}\ln\frac{\bar{q}}{1-\bar{q}}. \tag{87}$$

Then, averaging (87) over time T, we have that

$$\frac{1}{T}\int_0^T \bar{p}(1-\bar{p})dt = \frac{b}{\gamma} - \frac{1}{\gamma}\frac{1}{T}\left[\ln\frac{\bar{q}}{1-\bar{q}}\right]_0^T. \tag{88}$$

For a trajectory that remains in the interior, $\ln\frac{\bar{q}}{1-\bar{q}}$ is bounded, and so

$$\lim_{t\to\infty}\frac{1}{T}\left[\ln\frac{\bar{q}}{1-\bar{q}}\right]_0^T = 0. \tag{89}$$

Thus, we get that, for large time T,

$$\frac{1}{T}\int_0^T \bar{p}(1-\bar{p})dt = \frac{b}{\gamma} = \bar{p}^*(1-\bar{p}^*). \tag{90}$$

Similarly, from the expression for $\dot{\bar{p}}$ we get that

$$\frac{1}{T}\int_0^T \bar{q}(1-\bar{q})dt = \frac{a}{\beta} = \bar{q}^*(1-\bar{q}^*). \tag{91}$$

Initial conditions for slow-varying and non-averaged frequencies are not the same

We have that

$$p(0) = \bar{p}(0) + \epsilon\big(\bar{p}(0), \bar{q}(0), 0\big), \tag{92}$$

and from (46) and (39) we have that

$$\epsilon\big(\bar{p}(0),\bar{q}(0),0\big)=\frac{1}{\omega}G\big(\hat{A}(0),\hat{B}(0),\bar{p}(0),\bar{q}(0)\big)=\frac{1}{\omega}\bar{p}(0)\big(1-\bar{p}(0)\big)\Big(\hat{A}(0)-\hat{B}(0)\bar{q}(0)\Big). \tag{93}$$

Now, from the definition of $\hat{A}$, $\hat{B}$ in (47), it can be seen that the values of $\hat{A}(0)$ and $\hat{B}(0)$ are, in general, not necessarily zero, and hence we have that, in general, $\epsilon\big(\bar{p}(0),\bar{q}(0),0\big)$ need not be zero.

Hence, we have that in general $p(0)\neq\bar{p}(0)$, and so different initial conditions have to be input for the slow-varying and non-averaged frequencies. The discrepancy in initial conditions can be computed via solving the equation (93).
